# Validation of the Brazilian version of the pediatric outcomes data collection instrument: a cross-sectional evaluation in children and adolescents with juvenile idiopathic arthritis

**DOI:** 10.1186/1471-2431-13-177

**Published:** 2013-10-30

**Authors:** Felipe Alves do Monte, Moacir Novaes Lima Ferreira, Kátia Cristina Lima Petribu, Nair Cristina Almeida, José Benjamim Gomes, Maria Helena Mariano, Zelina Barbosa Mesquita, Diego Montarroyos Simões, André Furtado de Ayalla Rodrigues, Mariana Alves Nogueira Souza

**Affiliations:** 1 Clinical Research Unit of Oswaldo Cruz University Hospital and Pernambuco, Cardiologic Emergency at Pernambuco University, Rua Arnóbio Marques, 310, Santo Amaro, Recife, PE 52011-240, Brazil; 2 Pediatric Rheumatology Unit, Professor Fernando Figueira Institute of Integral Medicine, Rua dos Coelhos, 300, Coelhos, Recife, PE 50070-550, Brazil

**Keywords:** Quality of life, Questionnaires, Musculoskeletal diseases, Children, Adolescents, Validation studies, Brazil

## Abstract

**Background:**

There is a lack of health-related quality of life (HRQOL) questionnaires to evaluate pediatric musculoskeletal diseases in Brazil. The Pediatric Outcome Data Collection Instrument (PODCI) is widely used elsewhere for pediatric patients with musculoskeletal disorders, but it has not been fully validated in Brazil. Validation of the PODCI in the Brazilian Portuguese language is important to improve the assessment of pediatric patients with musculoskeletal diseases and to compare Brazilian study results with results from the international literature. This study aimed to analyze the test–re-test reliability and the convergent validity indicators for the quality of life scores obtained by application of the PODCI to children and adolescents with juvenile idiopathic arthritis (JIA).

**Methods:**

The PODCI underwent translation, transcultural adaptation, and field testing. Fifty-seven children and adolescents with JIA were administered the PODCI questionnaire. The Child Health Questionnaire - Parent Form 28 (CHQ PF-28) was used as the gold standard. Pain scales were employed, clinical examinations were performed, and laboratory inflammatory activity tests were conducted.

**Results:**

The three versions of the PODCI exhibited good internal consistency (Cronbach’s alpha coefficient >0.70), good reproducibility (p < 0.05), and good correlation compared with the gold standard (CHQ), as shown by a Spearman coefficient (Rho) >0.40 (p < 0.05).

**Conclusions:**

The PODCI was validated in patients with JIA in Brazil. This questionnaire was found to be valid, precise, and reliable. It can be successfully applied in research conducted by healthcare professionals who work with children and adolescents with musculoskeletal system disorders.

## Background

Advances in health sciences have improved the methodologies used to analyze the outcomes of clinical and surgical treatments. Measurements, such as health-related quality of life (HRQOL), functional capacity, pain scores, and personal satisfaction scales, have been widely employed because they capture the concerns of individuals and provide a measure of the burden of disease for individuals and of the effects of treatment.

Health status questionnaires such as the Child Health Questionnaire (CHQ) [1], the Pediatric Quality of Life Inventory (PedsQL) [2] and the Pediatric Outcome Data Collection Instrument (PODCI) [3] are used worldwide in pediatric patients with a variety of acute and chronic conditions (e.g., asthma, sleep apnea, neuromuscular diseases) and also in those with musculoskeletal diseases. CHQ and PedsQL monitor generic health status while the PODCI covers functionality of special significance to individuals with musculoskeletal impairments such as orthopedic and rheumatologic conditions [4-12].

Most HRQOL questionnaires are available in English. Whenever an adequately validated measurement assessing a given condition exists in any language, it is time-saving and economically advantageous to validate an existing instrument in a different language rather than develop a new instrument [13], and guidelines are available to establish the transcultural equivalence of such questionnaires [14,15].

There are several reasons to validate the PODCI in Brazil. These include its wide range of functional measurements, its use in international publications [4-11], the multidisciplinary construction of the questionnaire, and the possibility of its use by professionals in different areas of research. It is a sensitive instrument for detecting changes in the health states of individuals [3] and is considered the most comprehensive instrument for use in children, adolescents and caregivers [12,16]. Therefore, the aim of this study was to analyze and validate the reproducibility and the convergent indicators of QOL scores obtained by the PODCI in children and adolescents with juvenile idiopathic arthritis (JIA) in Brazil.

## Methods

The study was approved by the Human Research Ethics Committee of Oswaldo Cruz University Hospital/Pernambuco Cardiologic Emergency Room of Pernambuco University (protocol – Research Ethics Committee: no. 10/2010). The study population comprised patients attending the pediatric rheumatology outpatient clinic of the Professor Fernando Figueira Institute of Integral Medicine.

A convenience sample was used and the size was estimated by assuming a Spearman’s correlation coefficient of 0.60 from the results of a previous study [17] in which PODCI measurements were correlated with those of the CHQ Parent Form-28 (CHQ PF-28). Furthermore, the sample size was supported by a previous study of the evaluation of the PODCI by Daltroy et al. [3]. Hence, assuming a statistical power of 80% and a significance level of 95%, the minimum sample size was estimated to be 19 JIA patients. The sample consisted of children and adolescents of both sexes who were diagnosed with JIA according to the International League of Associations for Rheumatology diagnostic criteria [18-20], and had no other physical and/or mental comorbid conditions.

The patients’ caregivers were informed of the aims of the study and of the possible risks and benefits, and were asked to sign an informed consent form (participation was voluntary). The PODCI and CHQ PF-28 questionnaires were completed by the patients’ caregivers between September and December 2010. An experienced rheumatologist in our group assessed disease activity as one of four levels: (0) no activity, (1) mild, (2) moderate, and (3) severe [21].

Data on HRQOL were collected by administration of the PODCI and the CHQ PF-28. Three versions of PODCI questionnaires were used: Q1 was given to parents or caregivers of patients who were 2 to 10 years old; Q2 was given to parents or caregivers of individuals who were 11 to 18 years old; and Q3 was given to patients who were 11 to 18 years old. There were no major differences among the three versions of the PODCI. There were a few modifications to adapt the questionnaires to activities related to the age group and to the person interviewed (patient or caregiver).

The PODCI questionnaire consisted of 48 items encompassing six domains: 1) function of the upper limbs (eight items); 2) transfer and basic mobility (11 items); 3) sports and physical function (21 items); 4) comfort and pain (three items); 5) satisfaction with physical condition (five items); and 6) global function. Global function was represented by the mean scores of the first four domains listed above. Translation, transcultural adaptation, and validation of the PODCI followed the stages recommended by the American Academy of Orthopedic Surgeons (AAOS) [15,22]: forward translation, backward translation, evaluation by an expert committee, pre-testing, and field testing.

The CHQ PF-28 consisted of 14 domains. This instrument was applied by means of interviews with the patients’ caregivers. The physical function domain (three items) was used as the gold standard following the original process of the PODCI validation recommended by the AAOS/Pediatric Orthopedic Society of North America [3,22].

The dependent variables were the scores obtained for each separate domain and one PODCI global measurement of quality of life. Scores were graded between 0 and 100, where 0 was rated the worst and 100 was rated the best. The independent variables were functional dimension scores obtained by means of the CHQ PF-28, the Faces Pain Scale [23], and the visual analog scale [24,25], the disease activity score, the number of active joints, and the number of limited joints. The CHQ PF-28 scores were graded between 0 and 100. The number of active joints and limited joints were identified by the attending physician. Joints were rated as limited when they exhibited some motion deficiency and were rated as active when they were swollen or movement was limited by pain. Patients were divided into two categories according to age range: 2 to 10 years old (children) and 11 to 18 years old (adolescents).

Test–re-test reliability of the PODCI was obtained after an interval of 24–48 hours, and the intraclass correlation coefficient was determined. The convergent validity coefficient was estimated by comparing the CHQ physical function score and the PODCI final score. The internal consistency of the instrument was established by Cronbach’s alpha coefficient (α), where 0.70 was rated sufficient for validation studies [3].The significance level was established at 5% to reject the null hypothesis in each analysis.

Data were entered into a data file using EpiData v.3.1 software (EpiData, Odense, Denmark); electronic procedures were used to control data entry. Data analysis was performed using the statistical package SPSS for Windows v. 10 (IBM Corp., Armonk, NY, USA); descriptive (i.e., frequency distribution, central tendency measures, dispersion, and amplitude) and inductive statistical procedures were employed.

Dispersion graphics were used to determine the presence of outliers, which were excluded from the analysis. The Shapiro-Wilk test was used to analyze the normal distribution.

## Results

The validity study effectively assessed 57 patients (32 children and 25 adolescents). The mean age was 10.1 years old (standard deviation (SD), 4.3), 71.4% were female, and polyarticular JIA was the most frequent disease subtype (33.3%). The reproducibility study included 28 individuals (16 children and 12 adolescents). The mean age was 10.2 years old (SD, 3.9), and 64.3% were female. Descriptive data of the scores of the three versions of the PODCI and of the CHQ PF-28 physical function domain are presented in Table 1.

**Table 1:** Scores of the PODCI and the CHQ PF-28 physical function domains in children and adolescents with juvenile idiopathic arthritis

**Domain**	**n**	**Mean**	**Standard deviation**	**Median**	**Minimum**	**Maximum**
Q1 - Children						
Function of the upper limbs (PODCI)	31	78.36	20.88	83.33	33.33	100.00
Transfer and basic mobility (PODCI)	30	78.69	25.04	89.86	15.97	100.00
Sports and physical function (PODCI)*	31	60.62	29.48	66.84	2.78	100.00
Comfort and pain (PODCI)*	31	58.99	19.88	56.00	20.33	100.00
Happiness/satisfaction (PODCI)	22	65.59	29.94	72.50	0.00	100.00
Global function (PODCI)	30	69.03	18.69	75.81	29.67	91.67
Physical function (CHQ-PF 28)	32	56.25	36.11	66.67	0.00	100.00
Q2 - Adolescents (self-reported)						
Function of the upper limbs (PODCI)	23	91.30	12.04	95.83	58.33	100.00
Transfer and basic mobility (PODCI)	23	85.56	19.01	93.94	36.55	100.00
Sports and physical function (PODCI)*	23	57.18	28.50	50.00	10.22	100.00
Comfort and pain (PODCI)*	23	64.40	13.48	66.67	31.00	82.63
Happiness/satisfaction (PODCI)	23	75.65	22.38	80.00	15.00	100.00
Global function (PODCI)*	23	74.61	13.74	73.42	45.82	92.20
Q3 - Adolescents (reported by caregivers)						
Function of the upper limbs (PODCI)	25	81.07	24.22	95.24	20.83	100.00
Transfer and basic mobility (PODCI)	24	78.67	22.21	80.97	13.64	100.00
Sports and physical function (PODCI)	25	49.08	28.96	40.72	6.82	100.00
Comfort and pain (PODCI)	25	63.16	18.86	66.67	22.67	92.10
Happiness/satisfaction (PODCI)*	24	61.87	27.81	62.5	10.00	100.00
Global function (PODCI)*	24	68.24	16.33	68.91	37.48	92.70
Physical function (CHQ PF-28)	24	42.36	36.29	33.33	0.00	100.00

PODCI = Pediatric Outcome Data Collection Instrument; CHQ PF-28 = Child Health Questionnaire - Parent Form 28.

*Data follow a normal distribution.

The scores of some of the questionnaire domains could not be calculated because they had “lost data” according to the criteria recommended by the AAOS [23]. The number of lost domains varied between 0% and 8% in the three versions of the PODCI; the happiness/satisfaction domain, which was answered by the patients, exhibited a 50% data loss.

All three PODCI versions exhibited good internal consistency. The pediatric version had Cronbach’s α = 0.9, whereas adolescents who self-reported their answers and whose caregivers answered the questions had α values of 0.82 and 0.72, respectively.

Table 2 shows the results of the reproducibility of each domain of the three versions of the PODCI. The test–re-test intraclass correlation coefficient concordance varied between 0.69 and 0.97 in Q1, between 0.53 and 0.97 in Q2, and between 0.04 and 0.86 in Q3. All three versions exhibited good reproducibility in most questionnaire domains.

**Table 2:** Intraclass correlation coefficient (IC) of data collected during first and second administration of the PODCI

**Domain**	**Q1**		**Q2**		**Q3**	
	**Children**		**Adolescents (self-report)**		**Adolescents (reported by caregivers)**	
	**IC**	**p**	**IC**	**p**	**IC**	**p**
Physical function of the upper limb	0.89	<0.001	0.97	<0.001	0.86	<0.001
Transfer and basic mobility	0.97	<0.001	0.81	<0.001	0.04	0.449
Sports and physical function	0.97	<0.001	0.97	<0.001	0.60	0.014
Pain and comfort	0.82	<0.001	0.59	0.016	0.18	0.274
Happiness/satisfaction	0.69	0.001	0.53	0.032	0.68	0.006

Good correlation was observed (Rho ≥0.50) between the CHQ PF-28 physical function domain scores and global function in all three versions of the PODCI questionnaire (Table 3).

**Table 3:** Spearman’s correlation coefficients (Rho) comparing the CHQ PF-28 questionnaire physical function domains and global function in the three versions of the PODCI questionnaire

**PODCI (global function) × CHQ PF-28**	**Rho**	**p value**
Q1 - Children	0.79	<0.01
Q2 - Adolescents (self-reported)	0.67	<0.01
Q3 - Adolescents (reported by caregivers)	0.50	0.02

An inverse correlation was observed between the numbers of active and limited joints and the PODCI and CHQ PF-28 questionnaire scores (Table 4).

**Table 4:** Spearman correlation coefficients (Rho) comparing the PODCI global function scores, the CHQ PF-28 physical function scores, and the numbers of active and limited joints

**Questionnaires**	**Number of active joints**		**Number of limited joints**	
	**Rho**	**p**	**Rho**	**p**
Q1 - Children				
PODCI (global function)	-0.56	<0.01	-0.41	0.03
CHQ PF-28 (physical function)	-0.63	<0.01	-0.49	<0.01
Q2 - Adolescents (self-reported)				
PODCI (global function)	-0.51	0.01	-0.56	<0.01
Q3 – Adolescents (reported by caregivers)				
PODCI (global function)	-0.48	0.03	-0.57	<0.01
CHQ PF-28 (physical function)	-0.32	0.16	-0.58	<0.01

## Discussion

The main finding in this study was that all three versions of the PODCI exhibited excellent internal consistency and good reproducibility compared with the gold standard (CHQ); thus, the PODCI demonstrated a high level of precision. The psychometric properties were similar to those observed in the original PODCI elaboration and validation study [3]. A recent study validating the PODCI in Korean children observed that items in the pain/comfort and personal satisfaction domains exhibited low internal consistency [26], which was not observed in our study.

Response rates of only 50% in the happiness/satisfaction domain may have resulted from difficulties in understanding the questions in this domain during the interview, and the usefulness of this domain is questionable. However, this domain is not included in the measurement of “global function”, so missing data should not compromise the use and validity of the instrument.

In our study, the happiness/personal satisfaction domain exhibited low reproducibility in some versions and for some items of the questionnaire. This finding was also observed in a study of United States’ subjects [3]. Additionally, an item assessing sports activities at school, “How often in the last week did your child participate in gym/recess?”, exhibited low reproducibility. This was probably a result of the similarity between two possible answers, “school is not in session” and “my child does not attend school”. In both situations, items exhibiting low reproducibility were not statistically significant.

Assis et al. [27] previously validated the PODCI only in parents or caregivers in Brazil and observed the sensitivity of the PODCI for detecting changes in the health states of ill individuals. However, they did not validate this instrument for its capacity to detect QOL in a cross-sectional sample, and did not evaluate its precision.

As in the original PODCI validation study, the CHQ PF-28 physical function was used as the gold standard [3] to assess convergent validity. In our study, the validity of the PODCI translated into Portuguese was compared with the CHQ PF-28 physical function domain. All items exhibited good correlations with the gold standard. The inverse correlation between the number of affected joints, the PODCI, and the gold standard questionnaire scores support its convergent validity.

The PODCI measures cross-sectional or longitudinal QOL in individuals which distinguishes the PODCI from other generic questionnaires [3,26,27]. The sensitivity of the PODCI to clinical changes [3] makes it a valuable instrument [28], and the sensitivity of all three PODCI versions to changes in Brazilian patients was verified. Finally, PODCI reference values for healthy Brazilian individuals have not been established. Most of the generic questionnaires used in patients with JIA cannot be used for longitudinal assessments, making them unsuitable for evaluating treatment outcomes, whereas the PODCI is applicable for such assessments. However, the PODCI needs to be further validated in Brazil in longitudinal studies of treatment outcome.

## Conclusions

The PODCI was translated into Portuguese, and adapted and validated for use in Brazilian children and adolescents with JIA and their caretakers. This new questionnaire was determined to be valid, precise, and reliable and should be useful for studies in children and adolescents with musculoskeletal disorders.

## Abbreviations

HRQOL: Health-related quality of life; CHQ: Child health questionnaire; PedsQL: Pediatric quality of life; PODCI: Pediatric outcome data collection instrument; JIA: Juvenile idiopathic arthritis; CHQ PF-28: Child health questionnaire - parent form 28; AAOS: American academy of orthopedic surgeons.

## Competing interests

The authors declare that they have no competing interests.

## Authors’ contributions

FAM conceptualized and designed the study, analyzed the data and drafted the manuscript. FAM, MNLF, KCLP, NCA, JBG, MHM, ZBM and MHL provided comments and revisions to the manuscript. FAM and NCA participated in the translation and adaptation of the questionnaires. ZBM performed the clinical evaluation of all patients with JIA. FAM, DMS, AFAR and MANS collected and analyzed the data. All authors read and approved the final manuscript.

## Authors’ information

FAM, MNLF, KCLP, NCA, JBG, MHM, DMS, AFAR and MANS are researchers from the Clinical Research Unit, Oswaldo Cruz University Hospital/Pernambuco Cardiologic Emergency Room of Pernambuco University. ZBM is a doctor working at the Professor Fernando Figueira Institute of Integral Medicine.

## Pre-publication history

The pre-publication history for this paper can be accessed here:

http://www.biomedcentral.com/1471-2431/13/177/prepub
